# Effect of Production Technology Intensity on the Grain Yield, Protein Content and Amino Acid Profile in Common and Durum Wheat Grain

**DOI:** 10.3390/plants12020364

**Published:** 2023-01-12

**Authors:** Alicja Sułek, Grażyna Cacak-Pietrzak, Marcin Różewicz, Anna Nieróbca, Jerzy Grabiński, Marcin Studnicki, Katarzyna Sujka, Dariusz Dziki

**Affiliations:** 1 Department of Cereal Crop Production, Institute of Soil Science and Plant Cultivation—State Research Institute, 8 Czartoryskich Street, 24-100 Pulawy, Poland; 2 Department of Food Technology and Assessment, Institute of Food Sciences, Warsaw University of Life Science, 159C Nowoursynowska Street, 02-787 Warsaw, Poland; 3 Department of Agrometeorology and Applied Informatics, Institute of Soil Science and Plant Cultivation—State Research Institute, 8 Czartoryskich Street, 24-100 Pulawy, Poland; 4 Department of Biometry, Institute of Agriculture, Warsaw University of Life Science, 159 Nowoursynowska Street, 02-776 Warsaw, Poland; 5 Department of Thermal Technology and Food Process Engineering, University of Life Sciences in Lublin, 31 Głeboka Street, 20-612 Lublin, Poland

**Keywords:** common wheat, durum wheat, integrated technology, intensive technology, yield, protein amino acid profile, biological value of protein

## Abstract

Products derived from wheat grains are an important source of protein in the daily diet of people in many parts of the world. The biological value of protein is determined by its amino acid composition and the proportions of the individual amino acids. Synthesis of these compounds in wheat grains is influenced by genetic factors, as well as habitat conditions and the agrotechnology applied in cultivation. The aim of this study was to assess the effect of production technology (integrated, intensive) on the grain yield and the content amino acid profile of protein in common and durum wheat grain. Field research was conducted at the Experimental Station IUNG-PIB in Osiny (Poland) in two growing seasons. It was found that grain yield significantly depended on the weather conditions in the years of harvesting and genotype, but did not depend on the production technology. On the other hand, the protein content and their amino acid composition depended significantly on the production technology and genotype. A significantly higher content of protein substances was found in durum wheat grain. Increasing the intensity of production technology had a positive effect on the total protein content and the content of individual amino acids, both exogenous and endogenous. The amino acid limiting the biological value of protein contained in grains of both wheat species was lysine, and the deficiency of this amino acid was significantly lower in grain protein from intensive than integrated cultivation technology.

## 1. Introduction

Consumption of cereals covers about 30–40% of the recommended daily protein intake for humans, and one of the most important sources of cereal protein in the diet are wheat-based products [1,2,3]. Wheat (*Triticum*) is one of the most important cereal crops due to the chemical composition of the grain and its unique technological properties. On a global scale, about 70% of the harvest of the wheat grain is used for food purposes, and in Europe about 50% [4,5]. In 2020, the world wheat area was more than 219 million hectares, accounting for 30% of the cultivated area of all cereals, and the grain harvest was 760.9 million tons [6]. Several species of wheat are currently grown, but two are economically dominant—common wheat (*T. vulgare*) and durum wheat (*T. durum*)—which occupy 90% and 8–9% of the total wheat area, respectively [7,8]. In 2020, 119.2 million tons of common wheat grains and 7.5 million tons of durum wheat were harvested in European Union countries, representing 41.6% and 2.6%, respectively, of the total cereal harvest in the region [9].

Wheat is the most commonly used cereal for food purposes. For many years, there has been a high demand for high grain yield and good-quality grain on the world markets, especially for consumption. Therefore, intensive production technologies based on the extensive use of fertilizers and chemical crop protection products are commonly used. One of the most effective ways to quickly increase grain yield is the use of high doses of nitrogen fertilizer [10,11,12,13]. However, the intensification of production is costly, and long-term use leads to the degradation of the natural environment, e.g., soil, air, surface and groundwater pollution, and loss of ecosystem diversity [14]. An alternative to intensive production may be integrated technology, in which the use of crop protection products is limited to the necessary minimum, and the doses of mineral fertilizers are selected based on the results of soil tests and the assessment of the possibility of their use by a given plant species/cultivar. In such technology, it is possible to obtain a relatively high yield and good-quality grain while minimizing the negative influence on the natural environment and with increased efficiency of capital expenditures [15,16].

The production technology also affects the chemical composition of the grain, which consequently decides processing suitability and the nutritional value of the products obtained [10,11,12,17,18,19,20]. Cereal products provide a significant part of the energy from starch stored in grains, but also other nutrients, including protein, dietary fiber, macro- and microelements, and B vitamins [7,21,22].

The protein content in wheat grain is determined by many factors, including cultivar, growing conditions (climate, soil), agricultural technology and interactions between these factors [10,11,12,18,19,20,23,24,25,26,27,28,29]. The quality and content of protein determine the technological value of wheat grain and influence the quality and nutritional value of wheat products [30,31]. Amino acids are organic compounds constituting the building material of proteins. The most important factor determining the biological value of a protein is its amino acid profile and proportions in the content of individual amino acids, especially exogenous amino acids (tryptophan, arginine, lysine, histidine, leucine, valine, phenylalanine and methionine), which are not synthesized by the human body and therefore must be supplied with food [30,32,33,34]. Amino acids control blood sugar levels, renew the body’s energy resources, maintain good skin condition, strengthen bones, regenerate muscles and strengthen the immune and nervous systems [32]. The Food and Agriculture Organization (FAO) recommends treating amino acids as individual nutritional components of the diet, recommending paying attention not only to the amount of protein consumed but also to its amino acid profile [33]. Amino acid composition may change depending on wheat cultivar, habitat, fertilization, chemical protection and stress factors [32]. The aim of this study was to evaluate the effect of production technology (integrated and intensive) on the grain yield and the protein content of common and durum wheat grains, and their amino acid profile.

## 2. Results and Discusion

### 2.1. Grain Yield

**Figure 1:**
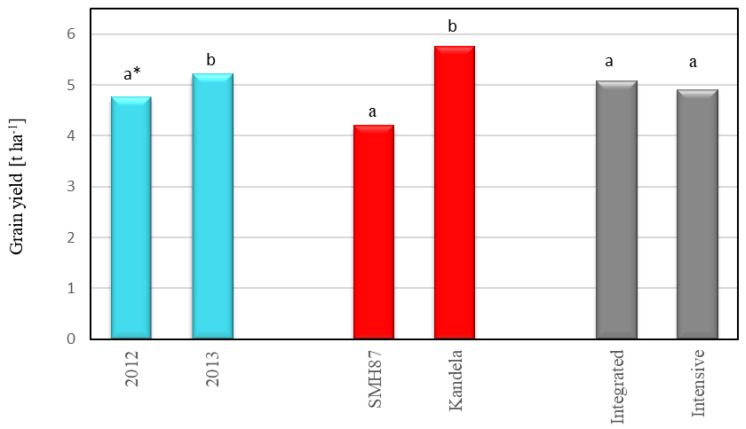
Wheat grain yield in relation to harvest year, cultivar and production technology. * Different letters (a, b) are significantly different (α = 0.05).

The genotype (species/cultivar) and weather conditions have an influence on the yield of wheat grain. In 2013, the grain yield was higher by 9.5% than in 2012 (4.76 and 5.21 t ha^−1^, respectively, Figure 1). Many studies [10,11,17,35,36,37] indicated that the decisive influence on the yield of spring wheat is the amount of precipitation, especially its distribution during the growing season. Due to the shallow root system of spring wheat, this species reacts particularly unfavorably to water deficiency and excess during tillering and stem elongation. Shortage of water in this period limits the growth of plant biomass. Excess water, on the other hand, limits the growth of roots, which has consequences in the later period, when rainfall shortages occur. Excess water also increases the occurrence of fungal diseases. Excessive rainfall during grain maturation and harvesting is particularly unfavorable, as it may, in addition to reducing the grain yield, cause deterioration of its quality, e.g., grain sprouting or contamination with mycotoxins [35,38]. In our research, higher yields were obtained in 2013, when the amount of precipitation in April and May was sufficient for the germination and wheat growth. High rainfall at the end of May and in June provided favorable conditions for plant growth and seed setting. Moderate rainfall in July, on the other hand, was conducive to the development of grain and its maturation on the main and side tillers.

This study showed that durum wheat yielded significantly lower than common wheat. The average durum wheat (cv. SMH87) grain yield was 4.20 t ha^−1^, which was 72.9% of the common wheat (cv. Kandela) yield (Figure 1). The obtained relationship is consistent with the results of research conducted by Rachoń and Woźniak [37]. They found that the grain yield of durum wheat in a 10-year period (2009–2018) was from 65.1% to 83.8% lower than common wheat. The most favorable proportions of hard wheat yield to common wheat yield were obtained in drier years (precipitation shortages in May and June), while the least favorable were in the vegetation period with the total precipitation in April–August exceeding 500 mm.

In this research, no significant effect of production technology on spring wheat grain yield was observed (Figure 1). Only a slight increase in the yield of wheat grain cultivated with the use of intensive technology was demonstrated (increase by 3.5%). Kołodziejczyk and Szmigiel [13] showed that the use of higher doses of nitrogen and fungicide protection contributed to an increase in the yield of common wheat grain by 1.49 t ha^−1^ (26.5%). In the studies of Sułek and Cacak-Pietrzak [11], it was shown that the use of intensive agricultural technology increased the yield of common wheat grain, but only in growing seasons with favorable weather conditions. Mariem et al. [17] showed that increased nitrogen fertilization applied from the flowering stage to milk ripeness had little effect on the durum wheat grain yield. Among the 20 durum wheat genotypes tested, two of them were characterized by higher grain yields with a lower level of nitrogen fertilization, while in the case of the other genotypes, with increasing the level of nitrogen fertilization, the yields were higher, but only slightly. The increase in grain yield resulted from the increase in the number of grains produced by the plant, but it was not correlated with the 1000 kernel weight.

### 2.2. Total Protein Content

**Figure 2:**
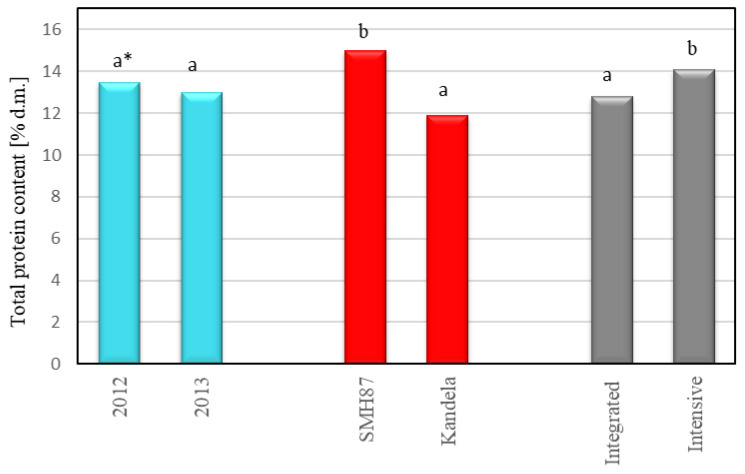
Total protein content of wheat grains in relation to harvest year, cultivar and production technology. * Different letters (a, b) are significantly different (α = 0.05).

The content of total protein in the grains of both common and durum wheat did not depend significantly on the course of weather conditions in the years of the study. However, a tendency to slightly higher synthesis of this component was found in grains harvested in 2012 compared to grains from 2013, respectively: 13.5% d.m. and 13.0% d.m. (Figure 2). A number of studies [23,36,39,40,41] show that weather conditions during the growing season, especially during flowering, grain filling, and grain ripening, influence the efficiency of nitrogen utilization by the plant and protein accumulation in grains. Michaletti et al. [42] showed that drought stress during flowering and grain filling reduces nitrogen utilization, leading to lower protein synthesis and lower grain content. Spychaj et al. [39] found that durum wheat grains had high total protein content in a year with very low precipitation (monthly sum of precipitation 12 mm) during the ripening period (month of July). In the study of Del Moral et al. [23], durum wheat grains grown in warm, dry climates with limited rainfall had a high protein content. Similarly, Kulyk et al. [40] showed a higher protein content in grains of winter common wheat cultivars when a deficit of precipitation was recorded during grain ripening.

In our study, the application of intensive production technology significantly increased the total protein content in wheat grains (Figure 2). The difference in the content of this component in wheat grains from intensive and integrated cultivation was 1.3 percentage points. A favorable effect of production intensification on total protein content in grains of spring wheat cultivars was also demonstrated in other works [17,18,19,20]. Literature data [10,17,20,28,43] indicated that the total protein content in wheat grains is favorably influenced by mineral nitrogen fertilization, especially when applied later in the vegetation period, i.e., at the heading stage. The application of nitrogen fertilization divided into two rates and the application of the second rate at the heading stage may increase the protein content in grains by as much as 7.5 percentage points [17]. On the other hand, the application of nitrogen in three/four applications improves the grain yield, but less effectively increases the protein content in grains (by 5.2 percentage points compared with a single dose) [20]. Mariem et al. [17] and Lollato et al. [44] found that the efficiency of nitrogen uptake by wheat is also significantly affected by genetic factors and interactions between factors, so the rate of nitrogen fertilization and the breakdown into individual doses should be adapted to the needs of the particular species/cultivar of wheat.

A significantly higher content of total protein was found in durum wheat (cv. SMH87) grains than in common wheat grains (cv. Kandela), respectively: 15.0% d.m. and 11.9% d.m. (Figure 2). A higher protein content in durum wheat grains than in common wheat grains was also indicated by Rachoń and Woźniak [37] and Geisslitz et al. [45]. Higher protein content in durum wheat grains is associated with the presence of genes that increase protein synthesis, which are however negatively correlated with grain yield [46]. Breeding programs aimed at increasing the protein content of wheat grain can be an effective tool for improving its quality in terms of this trait [24,47].

### 2.3. Protein Amino Acid Profile

**Figure 3:**
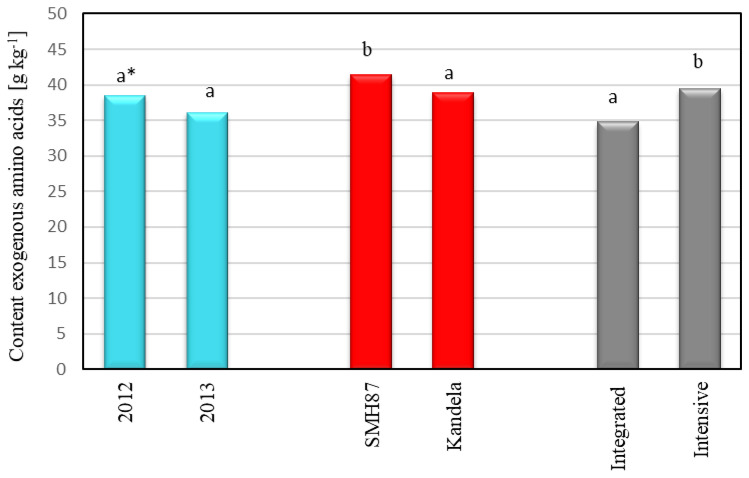
Content of exogenous amino acids in wheat grain depending on the year of harvest, cultivar and production technology. * Different letters (a, b) are significantly different (α = 0.05).

**Figure 4:**
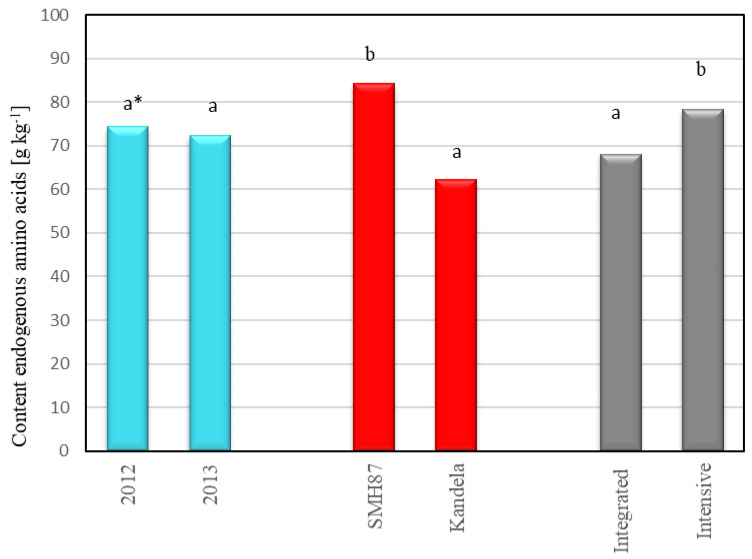
Content of endogenous amino acids in wheat grain depending on the year of harvest, cultivar and production technology. * Different letters (a, b) are significantly different (α = 0.05).

Analyzing the amino acid profile of protein, no significant effect of the year of harvest was found on the total content of exogenous amino acids or endogenous amino acids in wheat grains. However, there was a trend of slightly higher amino acid content in wheat grains from the 2012 harvest. The total content of exogenous amino acids in wheat grains from 2012 was on average 38.54 g kg^−1^, while in grains from 2013, it was 36.21 g kg^−1^ (Figure 3). Even smaller differences were found in the total content of endogenous amino acids. In the grains harvested in 2012, the content of endogenous amino acids was 74.21 g kg^−1^, while in grains from 2013, it was 72.20 g kg^−1^ (Figure 4). Numerous studies [23,24,41,48,49,50,51] show that the amino acid profile of wheat protein can be influenced by weather conditions during the growing season. Water availability in particular has a strong influence on the uptake of nitrogen by plants [23,41,48,49]. In the studies of Del Moral et al. [23], high temperatures and water availability shortened the time of filling of durum wheat grain, which caused an increase in the content of glutamine and proline and increased the content of gliadins that are rich in these amino acids. The amino acids whose amount depended most on weather conditions were tyrosine (26.4% variation), lysine (23.7% variation), methionine (20.3% variation), threonine (19.3% variation) and valine (15.6% variation). Jaśkiewicz and Szczepanek [52] found that increased rainfall in June-July, when grain filling and ripening took place, influenced the increase in amino acid content in winter triticale grains. Spychaj-Fabisiak et al. [51], studying the amino acid profile of winter wheat protein, showed that amino acid synthesis was favored by the abundant rainfall that occurred in July during grain ripening. According to Wan et al. [41], a shortage of precipitation and low water content in soil limit the uptake of nitrogen necessary for protein synthesis. Nitrogen is then extracted from leaves, especially from the flag leaf, which significantly reduces the amount of amino acids transported to grain, thus reducing the protein content and lowering the content of exogenous amino acids in it. The current research showed that the accumulation of a higher amount of amino acids in wheat grains was favored by favorable hydrothermal conditions during the vegetation period in 2012, in which high temperatures occurred during grain ripening, and at the same time, there were high moisture resources in the soil.

The grains of the spring wheat species/cultivars tested differed significantly in terms of the content of individual amino acids. The grains of durum wheat (cv. SMH87) contained 41.47 g kg^−1^ of exogenous amino acids and 84.25 g kg^−1^ of endogenous amino acids, while in grains of common wheat (cv. Kandela), the content of these compounds was, respectively, 38.82 and 62.18 g kg^−1^ (Figure 3 and Figure 4). Durum wheat (cv. SMH87) grains contained 20.2% more exogenous amino acids and 26.2% more endogenous amino acids than common wheat grains (cv. Kandela). Jiang et al. [24], Hospodarenko et al. [49], Dvořáćek et al. [53] and Knezevic et al. [54] showed that the content of amino acids in the grain of different cultivars of common wheat depends on genetic factors. Similarly, in research conducted by Biel and Maciorowski [55], the content of amino acids in the grains of different spring wheat cultivars was significantly differentiated (from 83.41 to 88.33 g kg^−1^). Spychaj-Fabisiak et al. [51] found no significant differences in the total amino acid content of winter wheat cultivars in grains.

Analyzing the effect of cultivation technology on the amino acid profile of wheat protein, it was found that significantly more amino acids, both exogenous and endogenous, were contained in grains from intensive than integrated cultivation. In wheat grains cultivated in intensive technology, the total content of exogenous amino acids was on average 39.49 g kg^−1^, and in grains from integrated farming 34.80 g kg^−1^ (Figure 3). The content of endogenous amino acids in wheat grains from integrated farming was 66.84 g kg^−1^, while in grains from intensive technology it was 78.33 g kg^−1^ (Figure 4). A significant effect of production technology on the amino acid content was also demonstrated by Jaśkiewicz and Szczepanek [52]. The authors, conducting research with winter triticale, found that the total content of amino acids in grains from intensive and integrated cultivation was, respectively: 41.19 and 39.02 g kg^−1^, while for endogenous amino acids, these values were, respectively, 68.98 g kg^−1^ for intensive technology grains and 65.63 g kg^−1^ for grains from integrated technology. Besaliev et al. [48] found that cultivation method (tillage, nonmoldboard loosening) had an influence on synthesis of amino acids in spring wheat grain. The application of nonmoldboard loosening increased the content of exogenous amino acids in wheat grains.

The amino acids present in the highest amount in the grains of both studied wheat species were glutamine and proline (Table 1), which are the basic amino acids of all fractions of cereal proteins, and in particular of storage proteins [54]. Statistical analysis showed the interaction of experimental factors (production technology and cultivar) in shaping the content of individual amino acids in wheat grain. The application of intensive production technology significantly increased the content of exogenous amino acids, such as threonine (by 11%), valine (by 23%), isoleucine (by 36%), leucine (by 23%) and lysine (by 30%) in the grain of durum wheat (cv. SMH87) compared to the content of these compounds in the grain from integrated technology (Table 2). In the case of common wheat (cv. Kandela), intensification of technology significantly increased only the content of leucine (by 15%). The application of intensive production technology significantly increased the content of most endogenous amino acids, except for cysteine, in the grain of durum wheat (cv. SMH87) (Table 1). The applied production technologies did not significantly differentiate the content of individual endogenous amino acids in grain protein of common wheat (cv. Kandela), except for serine, the content of which increased by 19.9% in grains from intensive cultivation. Many studies [52,56,57,58,59,60,61,62] indicate that the amino acid profile of cereal protein is influenced by the intensity of production technology. It is important to supply an adequate amount of nitrogen, but also the necessary micronutrients that support its conversion to amino acids. Fertilization with micronutrients contributes to an increase in the amino acid and total protein content of cereal grains. Isaychev et al. [62], applying fertilization with microelements (manganese, molybdenum, zinc, copper and cobalt) of spring and winter wheat obtained an increase in the content of exogenous amino acids: lysine by 85.7%, threonine by 116.7%, isoleucine + leucine by 20.9%, phenylalanine + tyrosine by 33.3% in comparison with the control variant. Similarly, Gondek et al. [60] showed a beneficial effect of copper and manganese availability on the amino acid composition of wheat protein, in particular on lysine content. Crista et al. [58] also found an increase in histidine content under the influence of applied zinc fertilization, but there was a decrease in isoleucine content. According to Zhang et al. [61], nitrogen fertilization has a beneficial effect on leucine and phenylalanine content. It also increases the arginine content [59]. Nowak et al. [63], in their study on the amino acid composition of spelt grain protein, found that the application of a nitrogen rate of 100 kg ha^−1^ resulted in a reduction in arginine, tyrosine and valine. On the other hand, the lack of influence of a higher agrotechnical level (application of higher nitrogen rates) on the amino acid content of spelt protein was reported by Biel et al. [64]. Jaśkiewicz and Szczepanek [52] showed that intensive production technology with higher nitrogen fertilization and intensive plant protection did not increase significantly the content of lysine and methionine, while within endogenous amino acids, nonsignificant differences were recorded for asparagine, proline, alanine, tyrosine and cysteine. In a field study with oats, Ralcewicz and Knapowski [65] found that the application of nitrogen in amounts up to 60 kg ha^−1^ caused an increase in the content of arginine and isoleucine in grains. However, in their field study with winter barley, Barczak and Nowak [57] showed that higher levels of agrotechnology also increased arginine and isoleucine, as well as lysine and methionine. Majcherczak et al. [56], in a study on winter barley, found that nitrogen rates of 120 and 180 kg ha^−1^ caused an increase in the proportion of glutamic acid and proline in total protein and a significant reduction in the other endogenous amino acids.

**Figure 5:**
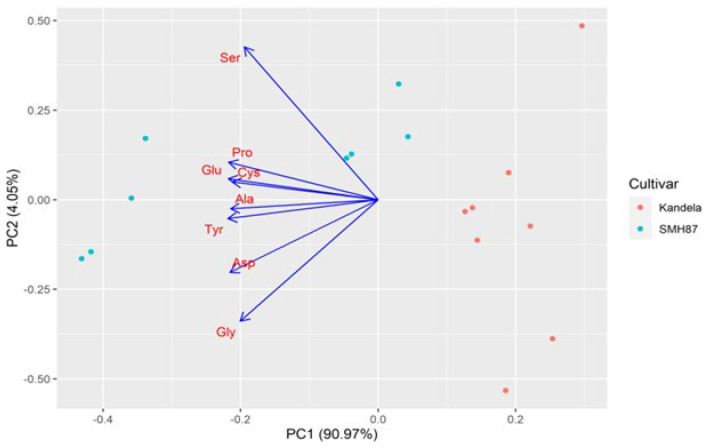
Biplot of principal component analysis for endogenous amino acid assessment results. Ser (serine), Asp (asparagine), Glu (glutamine), Pro (proline), Gly (glysine), Ala (alanine), Tyr (tyrosine), Cys (cysteine).

**Figure 6:**
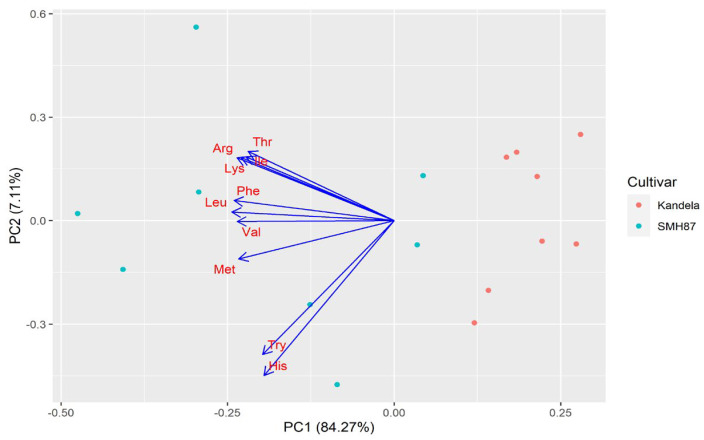
Biplot of PCA principal component analysis for exogenous amino acid assessment results. Thr (threonine), Val (valine), Ile (isoleucine), Leu (leusine), Phe (phenylalanine), His (histidine), Lys (lysine), Arg (arginine), Met (methionine), Try (tryptophan).

The graphs (Figure 5 and Figure 6) present the results of principal component analysis for the content of individual endogenous amino acids and exogenous amino acids in the grains of the tested wheat species/cultivars. A strong positive correlation was found between all the amino acids studied. The grain of durum wheat (cv. SMH87) in comparison with the grain of common wheat (cv. Kandela) had a higher content of all amino acids. The genetic factor (species/cultivar) had a greater share in the overall variability of both exogenous and endogenous amino acids than the production technology used. For exogenous amino acids, the effect of production technology was nearly twice as high as for endogenous amino acids (3% difference). However, the effect of genetic factor on the content of exogenous amino acids was lower than that for endogenous amino acids (by 6%).

Differences in the content of exogenous amino acids in wheat grain caused by genetic factors and applied production technology influenced the differences in the biological value of protein, evaluated on the basis of the value of the limiting amino acid (CS) index. The amino acid limiting the biological value of protein contained in the grain of both wheat species was lysine (Table 3). The protein contained in durum wheat (cv. SMH87) grains was characterized by a lower deficiency of this amino acid, with the deficiency being significantly lower in grain protein from intensive than integrated cropping technology. CS values for lysine in grain protein from integrated technology for both wheat species were at a similar level. However, common wheat (cv. Kandela) had a lower CS for lysine in grain protein from intensive than integrated technology (53% vs. 48%). In addition, there was also a deficiency in valine, with a higher CS for valine in grain protein from the intensive technology (93%) than from the integrated technology (88%). The grain protein of both wheat species tested had a higher biological value than in the study of Wiater and Kozera [66], in which, in addition to lysine, the amino acids limiting the biological value of wheat protein were isoleucine and valine. In the studies of Kowieska et al. [67], the amino acids limiting the biological value of wheat protein were lysine and threonine, in line with the FAO. In our study, higher CS values were characterized by the protein contained in the grains of durum wheat (cv. SMH87) than that of common wheat (cv. Kandela), confirming the results of Biel et al. [64] and Besaliev et al. [48], indicating a significant influence of genetic factors in shaping the biological value of cereal protein. Biel et al. [68] demonstrated that grain of naked oat cultivars is characterized, similarly to wheat grain, by a lysine deficiency.

In our study, for both wheat species/varieties, indices of essential amino acids EAAI_MH_ and EAAI_WE_ reached higher values for grains from intensive technology than from integrated technology (Table 4). As shown in the study by Zhang et al. [69], nitrogen fertilization influences the increase in EAAI values. Cultivation technology and the applied rate of nitrogen fertilization differentiate the values of both indices. As demonstrated by Barczak et al. [70], a high level of nitrogen fertilization may negatively influence the EAAI index through lowering the content of essential amino acids. Comparing the assessed indices, high EAAI_MH_ and EAAI_EC_ values were found for durum wheat grains of the SMH 78 cultivar grown in both technologies. Biel and Maciorowski [55] showed that the varietal factor has a greater influence on the values of EAAI_MH_ and EAAI_WE_ indices than the intensity of agrotechnical measures applied to the crop.

## 3. Materials and Methods

### 3.1. Site Characteristics, Experimental Design, and Agronomic Practices

Wheat grains came from field experiments conducted in 2012 and 2013 at the Agricultural Experimental Station Osiny (145 m n.p.m., φ = 51°47′ N, λ = 22°05′ E), belonging to the Institute of Soil Science and Plant Cultivation—State Research Institute in Pulawy. The experiment was established by the randomized sub-block method in three replications: on pseudo-oblitz soil classified as a good wheat complex and of bonitation classes II and IIIb. The soil was characterized by a neutral reaction (pH_KCl_ 6.77) and in 100 g, it contained 19.3 mg P_2_O_5_ and 16.3 mg K_2_O. The first factor was two production technologies—integrated and intensive. In the integrated system, doses of potassium and phosphate fertilizers were determined based on the content of these components in the soil. The total dose of nitrogen was established on the basis of the expected grain yield, soil conditions, and knowledge of the field, taking into account the type of forecrop and its fertilization. Specific nitrogen doses were refined on the basis of soil and plant tests. The size of the first dose was determined on the basis of the mineral nitrogen test (N_min_), which is a direct indicator of soil nitrogen available to plants. The size of the second and third dose was established based on the assessment of the nutritional status of the plants using plant tests. Protection against the occurrence of weeds, diseases and pests was carried out in accordance with the integrated method of reducing weed infestation and the perpetrators of diseases and pests [16].

Agrotechnical procedures applied in individual production technologies are presented in Table 5.

The second factor was the grain of two spring wheat species: common wheat of Kandela cultivar and durum wheat of SMH87 cultivar.

### 3.2. Meteorological Conditions

Weather data were obtained from the Agro-Meteorological Station of the Institute of Soil Science and Plant Cultivation located at the Experimental Station in Osiny, where field studies were conducted. The course of weather conditions was evaluated on the basis of decadal data: total precipitation (mm) and average air temperature (°C) measured 2 m above ground level. Weather conditions in the years of the study (2012–2013) were compared with the averages for the period 1971–2010.

**Figure 7:**
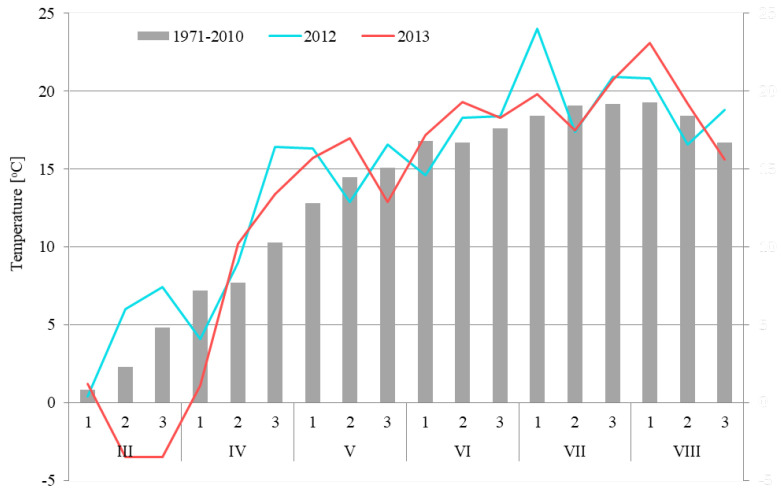
The average decade air temperature in year 2012–2013 and period 1971–2010.

**Figure 8:**
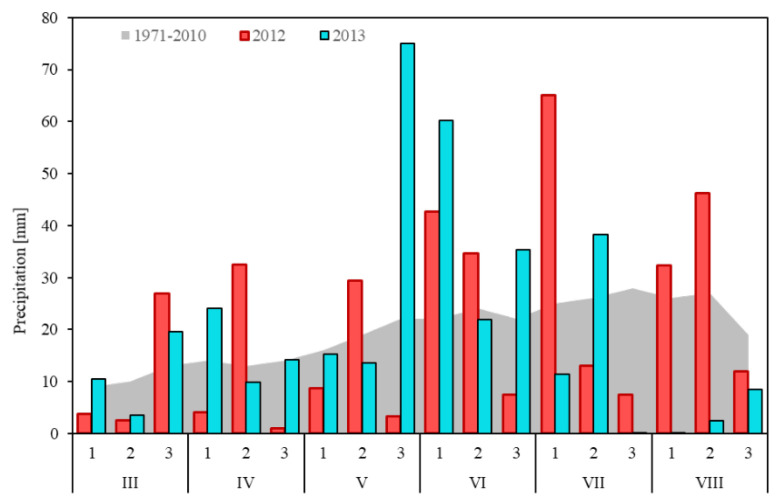
The average decade sum of precipitation in year 2012–2013 and period 1971–2010.

In the years of the study, weather conditions during spring wheat growth were differentiated. In 2012, the growing season was warmer than in 2013 (Figure 7), being characterized by a very warm March and extremely high air temperatures in the third decade of April (mean temperature 16.4 °C), which were higher on average by 6.1 °C compared to the mean decade temperature in the multiyear period 1971–2010. The next extremely warm period was the beginning of July (1st decade), where the mean air temperature was 24.0 °C, and was on average by 5.6 °C higher compared with the mean decade temperature for the multiyear period 1971–2010. At the same time, the year 2012 from the 3rd decade of April to the end of May saw moderate precipitation (Figure 8). Abundant precipitation occurred in the first two decades of June and at the beginning of July.

The year 2013 in terms of temperature was characterized by a late spring, with an average air temperature of −3.5 °C in the second and third decade of March (Figure 7). Such temperature conditions limited the spring vegetation of wheat. This very cold period was followed by rapid warming. The vegetation started to grow in the second decade of April and until the second decade of May the air temperatures were higher by 2.5–3.1 °C compared with the average temperatures recorded in the multiyear period 1971–2010. The change of weather conditions occurred at the turn of May and June. In this period, low air temperatures were recorded in the 3rd decade of May (mean temperature 12.9 °C), and simultaneously high precipitation occurred (mean precipitation 75 mm in the 3rd decade of May and 60 mm in the 1st decade of June) (Figure 8). The precipitation in these two decades constituted 307% of the average precipitation recorded in the years 1971–2010. After the period of abundant precipitation in May and June, at the turn of July and August the rain-free period occurred, which favored grain ripening.

### 3.3. Grain Yield Assessment

Wheat grain was harvested mechanically using a plot harvester at the stage of full grain maturity (BBCH 85). Grain yield per area unit was determined after harvesting.

### 3.4. Chemical Analyses

#### 3.4.1. Determination of Total Protein Content

The total protein content of the grains was determined using the Kjeldahl method (N 6.25) on a Kjeltec 8200 (Foss, Sweden) according to the methodology of AACC Method 46-11.02 [71].

#### 3.4.2. Identification and Determination of Amino Acids by High-Performance Liquid Chromatography (HPLC)

##### Apparatus

Determination was performed using the ACQUITY UPLC system chromatograph (Waters, Milford, MA, USA) equipped with a thermostat, autosampler, high-pressure binary pump, and PDA (an optical detector in the range ultraviolet-visible light that operates between 190 nm and 700 nm) and fluorescence detectors. Chromatographic separation was performed on AccQ-Tag Ultra C-18 (2.1 mm × 100 mm, 1.7 μm packing) and ZORBAX ODS C-18, (4.6 mm × 250 mm, 5 μm packing) columns.

##### Analytical Procedure for the Determination of Amino Acids

The determination of 17 amino acids (except tryptophan) was performed according to the methodology described by Szkudzińska et al. [72]. Chromatographic separation was performed on an AccQ-Tag Ultra C-18 column and quantified using a PDA detector at 260 nm. Amino acid identification was performed by comparing the retention times of the peak in the sample with that of the standard. The amino acid content of the sample was calculated using Empower software (Waters, Milford, MA, USA), using the internal standard.

##### Analytical Procedure for the Determination of Tryptophan Content

A 1 g sample (containing approximately 60 mg of pure protein) was placed in a 50 mL ampoule, and approximately 6 g of barium hydroxide octahydrate was added and thoroughly mixed. Then 13 mL of hot distilled water was added and mixed again. The ampoule was sealed over a gas burner. The samples were then hydrolyzed in an oven at 110 °C within 16–20 h. After cooling, samples were quantitatively transferred into 100 mL centrifuge tubes using 20 mL of hot distilled water, then 2 mL of α-methyl-tryptophan (2.5 µmol L^−1^) internal stock standard solution was added. The solution was acidified by adding 6 mL of hydrochloric acid (1 mol L^−1^). Next, 19.5 mL of sodium sulfate solution (1 mol L^−1^) was added to precipitate barium sulphate. The contents of the tubes were mixed and centrifuged for 20 min at 3000 rpm. The liquid from the precipitate was transferred to a 100 mL volumetric flask. The sediment was washed with 15 mL of hot distilled water and centrifuged again (15 min at 3000 rpm). The liquid from the precipitate was transferred to a 100 mL flask and 10 mL of methanol was added. The sample was acidified to pH 3 with hydrochloric acid. The pH value was controlled using a pH meter. Before dosing onto the HPLC column, the solution was filtered through a syringe filter. The chromatographic analysis parameters are shown in Table 6.

Tryptophan content was calculated according to the formula:

$$X=\frac{P_{Try_{pr}}}{P_{mTry_{pr}}}\cdot \frac{c_{mTry_{pr}}}{\bar{f\left(x\right)}}\cdot \frac{V_{k}\cdot f}{m}$$

where:

$$\bar{f\left(x\right)}=\frac{\sum_{i=1}^{n}f\left(x_{i}\right)}{n}$$

and:

$$f\left(x_{i}\right)=\frac{P_{Try_{wz}}}{P_{mTry_{wz}}}\cdot \frac{c_{mTry_{wz}}}{c_{Try_{wz}}}$$

X —free or total tryptophan content, in g kg^−1^,
$P_{Try_{pr}}$—peak area of tryptophan in extract or hydrolysate of test sample,
$P_{mTry_{pr}}$—peak area of α-methyl-tryptophan in an extract or hydrolysate of the test sample,
$c_{mTry_{pr}}$—concentration of the internal standard (α-methyl-tryptophan) in the extract or hydrolysate of the test sample, in µg mL^−1^,
$\bar{f\left(x\right)}$—average calibration factor,
$V_{k}$—final volume of extract or hydrolysate of sample, w mL,
f—dilution factor,
m—sample weight, in mg,
$\sum_{i=1}^{n}f\left(x_{i}\right)$—sum of the calibration factors for all titrations of tryptophan and α-methyl-tryptophan calibration solution during the analysis,
$f\left(x_{i}\right)$—calibration factor for a single calibration injection of the standard solution of tryptophan and α-methyl-tryptophan during the analysis,
n—number of rates of tryptophan and α-methyl-tryptophan calibration solution during the analysis,
$P_{Try_{wz}}$—peak area of standard tryptophan, calibration standard solution of tryptophan and α-methyl-tryptophan,
$P_{mTry_{wz}}$—peak area of internal standard, calibration standard solution of tryptophan and α-methyl-tryptophan,
$c_{mTry_{wz}}$—concentration of the internal standard in the calibration standard solution, in µg mL^−1^,
$c_{Try_{wz}}$—concentration of the tryptophan standard in the calibration standard solution, in µg mL^−1^.

#### 3.4.3. Determination of Biological Value of Protein

The biological value of the protein was determined on the basis of the so-called limiting amino acid (CS) index, using chicken egg white as a standard. The limiting amino acid (CS) was calculated by comparing the amount of individual exogenous amino acids contained in the studied protein (ai) with their content in the standard protein (as), according to the formula [34]:

$$\mathrm{CS}=\frac{\mathrm{ai}}{as}\times 100\%,$$

where:

ai—The exogenous amino acid content of the tested protein,
*as*—Exogenous amino acid content of the reference protein.

The exogenous amino acid index EAAI was calculated according to the methodology given by Tome [73] as the geometric mean of all exogenous amino acids to the content of these amino acids in a given standard:

$$EAAI={\left(\frac{\left(EAA1\times EAA2\ldots EAAn\right)\left[samples\right]}{\left(EAA1\times EAA2\ldots EAAn\right)\left[egg\right]}\right)}^{1/n}$$

This indicator is determined with reference to egg white (EAAI _WE_) [74] and the exogenous amino acid requirements of an adult (EAAI _MH_) [34].

### 3.5. Statistical Analysis

The obtained results were statistically processed in Statistica ver. 13.1 (StatSoft, INC., Tulsa, OK, USA) using Microsoft^®^ Excel 2020, Microsoft 365 software package (Addinsoft, Inc., Brooklyn, NY, USA). In order to compare the influence of the studied factors on the total protein content and its amino acid profile, ANOVA was applied, and the differences found were estimated with the Tukey test at the significance level of α = 0.05. Additionally, in order to determine to what extent the studied wheat grain samples differed from each other and which of the analyzed factors had the greatest influence on it, a principal component analysis (PCA) of the obtained results was performed.

## 4. Conclusions

The grain yield significantly depended on the course of weather conditions and the wheat genotype, whereas the intensity of the production technology did not affect the yield. Common wheat (cv. Kandela) yielded significantly higher than durum wheat (cv. SMH87). The total protein content in wheat grains and their amino acid composition were significantly affected by genotype and intensity of production technology. Significantly higher protein content was found in durum wheat grains than in common wheat. Genotype (species/cultivar) had a greater share in the total variability of both exogenous and endogenous amino acids than the applied production technology. For exogenous amino acids, the effect of production technology was nearly twice as high as for endogenous amino acids. However, the influence of genetic factors on the content of exogenous amino acids was lower than for endogenous amino acids. The amino acid limiting the biological value of protein contained in the grains of both wheat species was lysine. The protein contained in durum wheat (cv. SMH87) grains was characterized by a lower deficiency of this amino acid, with the deficiency being significantly lower in grain protein from intensive than integrated cultivation technology. Taking into account the lack of a significant impact of production intensity on the increase in yield in grain from integrated cultivation and the protein content suitable for processing, the intensification of the production technology of common wheat (cv. Kandela) was ineffective. However, the use of intensive production technology during the cultivation of durum wheat (cv. SMH87) is more justified due to the significant increase in the total protein content and the possibility of improving its amino acid profile.

## Figures and Tables

**Table 1:** Effect of production technology on the content of endogenous amino acids (g kg^−1^) in durum wheat and common wheat grains.

Production Technology	S *	K **	S	K	S	K	S	K	S	K	S	K	S	K	S	K
	Endogenous Amino Acids															
	Ser ***		Asp		Glu		Pro		Gly		Ala		Tyr		Cys	
Integrated	5.56 ^b^ ****	4.88 ^a^	5.36 ^b^	4.79 ^a^	38.77 ^b^	31,87 ^a^	12.20 ^b^	9.39 ^a^	3.80 ^b^	3.63 ^a^	3.56 ^b^	3.35 ^a^	2.10 ^a^	1.80 ^a^	2.74 ^a^	2.29 ^a^
Intensive	6.82 ^a^	4.07 ^b^	6.69 ^a^	4.88 ^a^	50.50 ^a^	32.49 ^a^	15.11 ^a^	9.63 ^a^	4.50 ^a^	3.76 ^a^	4.65 ^a^	3.24 ^a^	2.71 ^a^	1.84 ^a^	3.16 ^a^	2.45 ^a^

S *—SMH87 (durum wheat), K **—Kandela (common wheat), Ser ***—(serine), Asp (asparagine), Glu (glutamine), Pro (proline), Gly (glysine), Ala (alanine), Tyr (tyrosine), Cys (cysteine). **** Mean values indicated in the columns by different letters (a, b) are statistically significantly different (α = 0.05).

**Table 2:** Effect of production technology on the content of exogenous amino acids (g kg^−1^) in durum wheat and common wheat grains.

Production Technology	S *	K **	S	K	S	K	S	K	S	K	S	K	S	K	S	K	S	K	S	K
	Exogenous Amino Acids																			
	Thr ***		Val		Ile		Leu		Phe		His		Lys		Arg		Met		Trp	
Integrated	3.28 ^b^ ****	3.05 ^a^	4.31 ^b^	3.40 ^a^	4.03 ^b^	3.48 ^a^	7.34 ^b^	5.48 ^b^	5.19 ^a^	4.81 ^a^	2.34 ^a^	2.19 ^a^	2.45 ^b^	2.26 ^a^	4.41 ^a^	3.73 ^a^	2.99 ^a^	2.36 ^b^	1.4 ^a^	1.3 ^a^
Intensive	3.65 ^a^	2.99 ^a^	5.30 ^a^	3.63 ^a^	5.46 ^a^	3.58 ^a^	8.96 ^a^	6.30 ^a^	6.03 ^a^	4.37 ^a^	2.57 ^a^	2.51 ^a^	3.15 ^a^	2.16 ^a^	5.24 ^a^	4.05 ^a^	3.28 ^a^	2.83 ^a^	1.5 ^a^	1.4 ^a^

S *—SMH87 (durum wheat), K **—Kandela (common wheat). Thr *** (threonine), Val (valine), Ile (isoleucine), Leu (leusine), Phe (phenylalanine), His (histidine), Lys (lysine), Arg (arginine), Met (methionine), Trp (tryptophan). **** Values indicated in the columns by different letters (a, b) are statistically significantly different (α = 0.05).

**Table 3:** Limiting amino acid index values CS (%) as a function of production technology in cultivar.

Amino Acid	FAO/WHO * Amino Acid Composition of the Egg White (mg g^−1^)	CS (%)			
		SMH87 (Durum Wheat)		Kandela (Common Wheat)	
		Integrated	Intensive	Integrated	Intensive
Isoleucine	3.01 *	135	180	115	119
Leucine	5.30	140	169	118	119
Lysine	4.50	54	70	53	48
Methionine + cysteine	2.21	259	291	210	217
Tyrosine + phenylalanate	3.81	191	236	160	163
Threonine	2.30	143	159	152	130
Tryptophan	0.61	238	248	220	230
Valina	3.9	111	136	88	93

* Value assumed as 100%.

**Table 4:** Values for exogenous amino acids EAAI (%) depending on the production technology and cultivar.

Cultivar	SMH87 (Durum Wheat)		Kandela (Common Wheat)	
Production technology	integrated	intensive	integrated	intensive
EAAI _MH_	93.9	96.8	88.7	91.6
EAAI _WE_	62.6	64.5	59.1	61.1

**Table 5:** Characterization of applied technologies for wheat production.

Production Technology	Fertilization (kg ha^−1^)			Herbicides	Fungicides	Insecticides	Retardants
	N	P_2_O_5_	K_2_O				
Integrated	110 *	70 **	105 **	Mustang 306 SE (florasulan) 0.6 L ha^−1^, Axial 100 EC (pinoxaden) 0.4 L ha^−1^	Input 460 EC (prothioconazole, spiroxamine) 1.0 L ha^−1^	Fury 100 EW (zeta-cypermethrin) 0.1 L ha^−1^	-
Intensive	140 *	80 **	100 **	Mustang 306 SE (florasulan) 0.6 L ha^−1^, Axial 100 EC (pinoxaden) 0.4 L ha^−1^	Amistar 250 SC (azoxystrobin) 0.6 L ha^−1^ Artea 330 EC (propiconazole + cyproconazole) 0.5 L ha^−1^	Fury 100 EW (zeta-cypermethrin) 0.1 L ha^−1^	Modus 250 EW (ethyl trinexapac) 0.4 L ha^−1^

* The first rate of nitrogen was applied before sowing wheat in the amount of 50 kg ha^−1^ (integrated technology) and 60 kg ha^−1^ (intensive technology). The second dose of nitrogen was applied at the shooting stage in the amount of 40 kg ha^−1^ (integrated technology) and 50 kg ha^−1^ (intensive technology), and the third at the wheat earing stage in the amount of 20 kg ha^−1^ (integrated technology) and 30 kg ha^−1^ (intensive technology). ** Phosphorus and potassium were applied once before wheat sowing.

**Table 6:** Chromatographic analysis parameters for the determination of tryptophan.

Column Temperature	25 °C
Moving phase	A mixture of 3.00 g acetic acid, 900 mL distilled water and 50.0 mL of 1,1,1-trichloro-2-methyl-2-propanol solution. The mixture was brought to pH 5 using sodium hydroxide solution. The pH value was controlled with a pH meter. The mixture was then made up to 1 L with distilled water.
Flow rate	1 mL min^−1^
Detection wavelength	Excitation: = 280 nm, emission: = 356 nm,
Volume to be dosed	20 µL

## Data Availability

The data presented in this study are available on request from the first author.
